# Thyrotoxicosis due to Hashimoto's disease triggered by radiofrequency ablation for low-risk papillary thyroid microcarcinoma: A case report

**DOI:** 10.1016/j.radcr.2025.01.039

**Published:** 2025-01-21

**Authors:** Van Bang Nguyen, Hau Nguyen Van Vy, Binh Le Trong, Chi Van Le

**Affiliations:** a Department of Internal Medicine, Hue University of Medicine and Pharmacy, Hue University, Hue, Vietnam; b Department of Radiology, Hue University of Medicine and Pharmacy, Hue University, Hue, Vietnam; c Center of Endocrinology and Diabetes, Family Hospital, Da Nang, Vietnam

**Keywords:** Low-risk papillary thyroid microcarcinoma, Thyroid autoimmune disease, Radiofrequency ablation, Thyroid nodules, A case report

## Abstract

This paper reports a case of acute thyrotoxicosis (Hashitoxicosis) in a 48-year-old woman following RFA for a low-risk papillary thyroid microcarcinoma. This case highlights the importance of considering preprocedural thyroid antibody testing to anticipate potential autoimmune reactions. Further studies are recommended to clarify the role of such testing in predicting adverse immune responses following thyroid RFA.

## Introduction

In recent years, radiofrequency ablation (RFA) has gained attention as a minimally invasive treatment option for malignant, autonomously functioning, and benign symptomatic thyroid nodules [[1], [2], [3]]. The RFA procedure typically employs 2 key techniques: the moving-shot technique and the trans-isthmic approach, both guided by ultrasonography after local anesthesia with 2% lidocaine at the needle puncture site and thyroid capsule [2,4]. Despite the low complication rate of RFA, a range of complications can still arise. In one study, 48 complications (3.3%) were documented, including major complications (e.g., voice changes and tumor rupture), as well as minor ones (e.g., hematoma and skin burns), most of which resolved spontaneously [5]. Additionally, thyrotoxicosis from transient thyroiditis has been identifed as a risk following RFA, likely due to direct tissue trauma and subsequent release of thyroid hormones. Cases of hyperthyroidism associated with Graves' disease have also been reported following radiofrequency ablation [6].

This report presents a case of Hashitoxicosis in a healthy 48-year-old female who underwent RFA for low-risk papillary thyroid microcarcinoma (PTMC) at an outpatient clinic. This report would provide endocrinologists, surgeons, and radiologists with significant evidence to identify and manage thyrotoxicosis in patients with thyroid nodules being treated with RFA.

## Case presentation

**Fig. 1:**
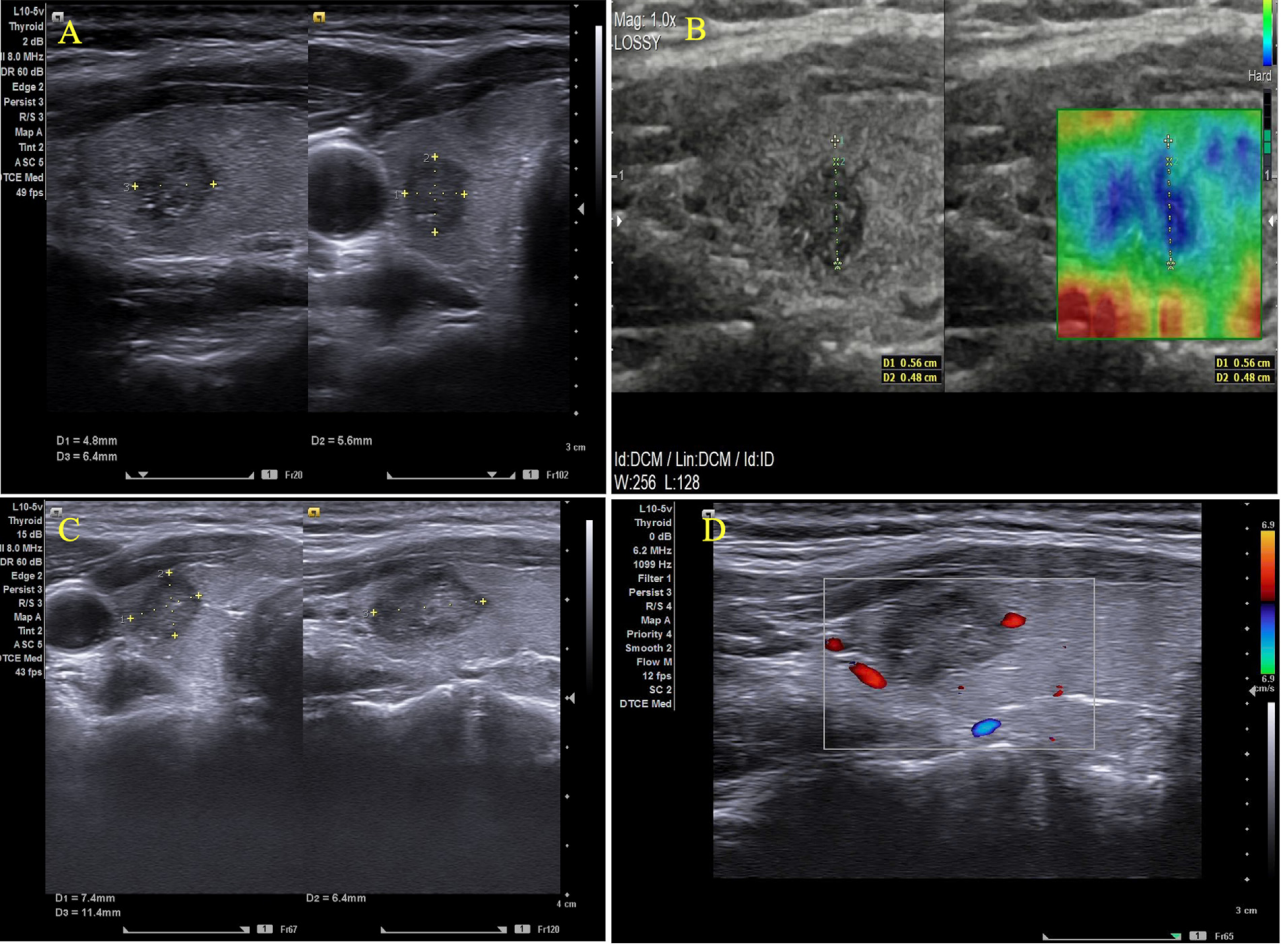
A 48-year-old woman presented to an endocrinologist following the incidental detection of a small thyroid nodule. (A) Thyroid ultrasound shows an ACR-TIRADS 5 nodule in the right lobe, measuring 4.8 × 5.6 × 6.4 mm. (B) Elastography reveals the entire lesion in hard blue with a strain ratio of 2.5. (C, D) A necrotic area measuring 7.4 × 6.4 × 11.4 mm is visible, with the surrounding thyroid tissue showing homogeneous echogenicity.

A 48-year-old woman, with no notable personal or family medical history, was referred to an endocrinologist following the incidental discovery of a small thyroid nodule through general check-up. Physical examination revealed no enlargement of the thyroid gland or palpable lymph nodes in the neck. Thyroid ultrasound identified a ACR-TIRADS 5 nodule in the right lobe, measuring 4.8 × 5.6 × 6.4 mm, with no suspicious lymph nodes or evidence of extrathyroidal extension (Fig. 1A) and the whole lesion appeared hard on elastography, with a strain ratio of 2.5 (Fig. 1B). A fine needle aspiration biopsy was performed, yielding a Bethesda VI classification on cytology, indicative of malignancy. Contrast-enhanced neck and lung CT scans showed no metastatic lesions or extrathyroidal extension. With aforementioned symptoms, the patient was subsequently diagnosed with low-risk PTMC.

The patient was suggested with 3 management options: surgery, minimally invasive treatment (RFA), and active surveillance. She expressed a desire for an active intervention rather than active observation, thus ruling out active surveillance. The patient declined lobectomy due to her concerns about potential hypothyroidism, voice changes, and cosmetic effects. For those reasons, she decided to undergo RFA for the nodule after signing the informed consent form.

Before the procedure, thyroid function tests - including FT4, thyrotropin (TSH), and additional tests such as complete blood count (CBC), liver and renal function tests, and prothrombin time - were all within normal ranges. Specifically, serum FT4 was 1.24 ng/dL (normal range [NR] 0.93-2.22), thyrotropin was 0.98 µIU/mL (NR 0.27-4.2), and thyroglobulin was 0.04 ng/mL (NR 3.5 - 77 ng/mL). The RFA procedure lasted 4 minutes and used 2 primary techniques, namely the trans-isthmic approach and the moving-shot technique. At a one-month follow-up, the clinical examination was unremarkable, and ultrasound showed a necrotic area measuring 7.4 × 6.4 × 11.4 mm with homogeneous echogenicity of the thyroid gland (Figs. 1C, 1D). FT4 and TSH remained within normal limits (TSH 1.04 µIU/mL, FT4 1.15 ng/dL).

**Fig. 2:**
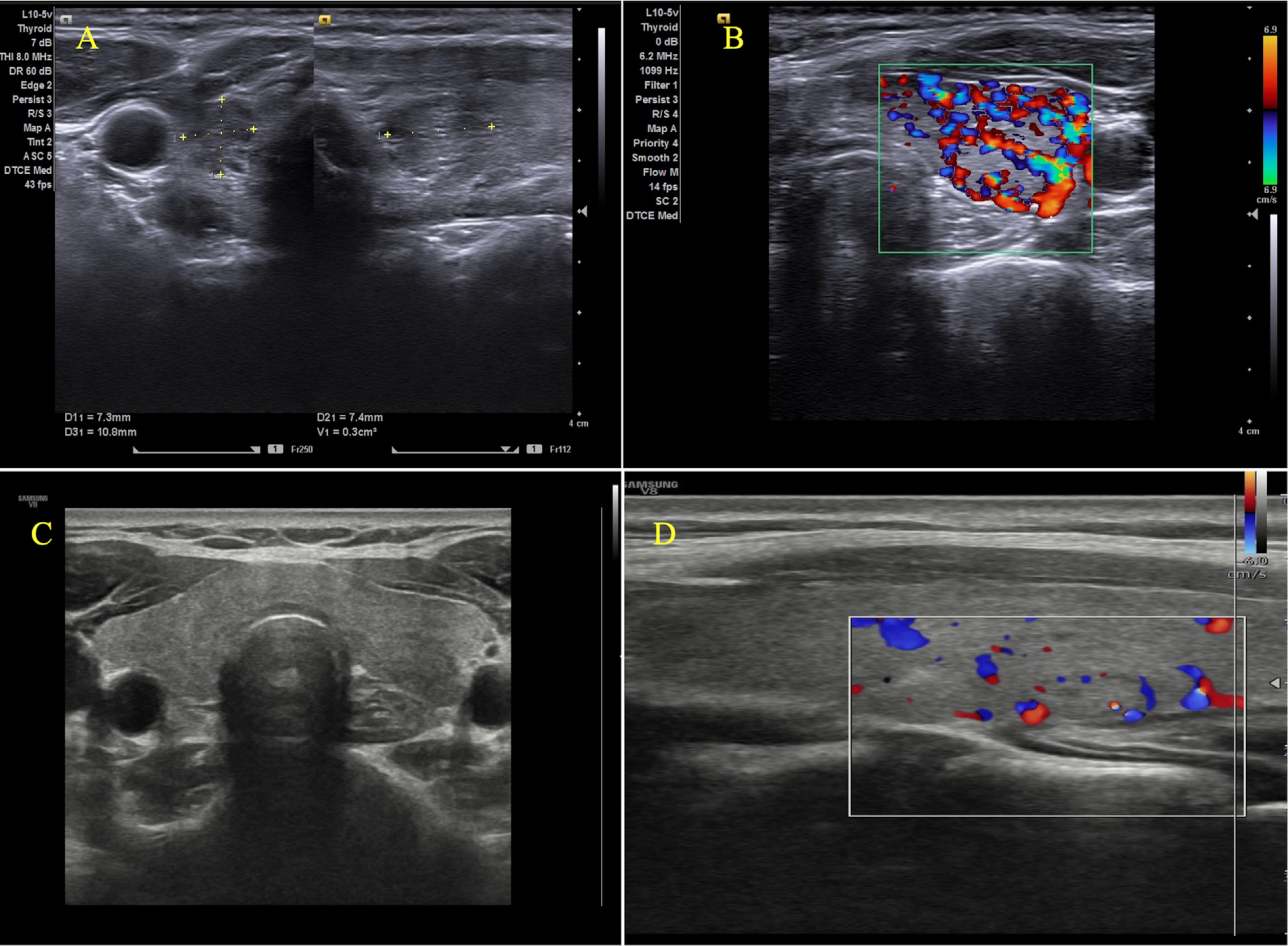
(A, B) Six months post-ablation, ultrasound demonstrates heterogeneous echogenicity in the thyroid gland with markedly increased vascularity on Color-Doppler imaging. (C, D) At 12 months post-ablation, ultrasound reveals homogeneous echogenicity of the thyroid gland with no signs of tumor recurrence.

However, 6 months after the ablation, the patient developed thyrotoxic symptoms, including weight loss and palpitations, though the neck examination remained normal. Ultrasound revealed heterogeneous echogenicity of the thyroid gland with increased blood flow on Color Doppler imaging (Figs. 2A, 2B). Blood tests showed a low TSH level (0.01 µIU/mL), elevated FT4 (3.35 ng/mL), high thyroid peroxidase antibody (TPO Ab) (383.3 IU/mL, NR < 34 IU/mL), elevated thyroglobulin antibody (TgAb) (437 IU/mL, NR < 115 IU/mL), and normal thyroid-stimulating immunoglobulin (TSI) (0.12 IU/mL, NR < 0.1 IU/mL). She was diagnosed with Hashitoxicosis following radiofrequency ablation and was treated with a beta-blocker to alleviate the relevant symptoms.

After 3 months of treatment, all of the symptoms resolved. At a 12 months post-ablation follow up, she showed no signs of thyrotoxicosis. Ultrasound revealed no tumor recurrence, with homogeneous echogenicity, reduced blood flow on Color Doppler imaging of the thyroid gland (Figs. 2C, 2D), and blood tests were in normal ranges.

## Discussion

In literature, minimally invasive techniques like RFA are widely recommended for PTMC treatment alongside lobectomy and active surveillance, as outlined in recent guidelines [1]. Studies have shown that RFA achieves complete disappearance rates of 98.8% and 100% at 24 and 60 months of follow-up, respectively. During the follow-up period, no local tumor progression, lymph node metastasis, or distant metastasis was observed, and none of the patients required delayed surgery. The rate of major complications was low (1.4%), with no delayed complications or procedure-related deaths reported [7]. Additionally, ultrasound-guided RFA has been associated with better quality-of-life outcomes compared to lobectomy [8].

With the case reported in this paper, a woman with right-sided PTMC opted for RFA after declining thyroidectomy. Six months post-ablation, she developed acute hyperthyroidism. Ultrasound and blood tests, combined with the absence of specific treatment, suggested Hashimoto's thyroiditis as the underlying cause. Although rare, cases of autoimmune thyroid disease triggering hyperthyroidism following minimally invasive thyroid procedures have been documented. Incident Graves' disease following percutaneous ethanol ablation has been reported [9], and a case of hyperthyroidism due to Graves' disease after RFA was described by Elizabeth et al. [6]. These cases suggest a mechanism involving thyroid trauma, the release of thyroid antigens, and the subsequent generation of TPO antibodies linked to Hashimoto's thyroiditis or thyrotropin receptor antibodies (TSI) associated with Graves' disease.

In the reported case, the hyperthyroid phase of Hashimoto's thyroiditis (Hashitoxicosis) has been differentiated from Graves' disease based on elevated TPO antibody levels and low TSI, the latter being specific for Graves' disease. Trauma to the thyroid parenchyma during RFA likely triggered an autoimmune response. Hashitoxicosis is typically a transient hyperthyroid phase of Hashimoto's thyroiditis that is self-limiting and generally resolves within weeks to months without specific treatment [10].

However, there are a number of limitations in establishing causality in this case. A significant limitation is the absence of preprocedural TSI and TPO antibody measurements, as initial ultrasound and thyroid function tests (TSH, FT4) showed no signs of thyroid autoimmunity prior to RFA. Routine testing for thyroid antibodies, including TSI, TRAb, or TPO antibodies, before performing RFA on benign or malignant thyroid lesions remains a topic of debate [11]. Failing to assess thyroid antibody levels before the procedure could lead to potential complications or delayed recognition of underlying autoimmune thyroid diseases, such as Graves' disease or Hashimoto's thyroiditis. The potential adverse effects of RFA, particularly those linked to thyroid autoimmune disease, might be under-recognized or poorly understood. Revisiting the utility of preprocedural antibody testing, including TSI, TRAb, and TPO levels, could help identify patients at risk and improve clinical outcomes. Future research would explore the cost-effectiveness and predictive value of incorporating these tests into routine preprocedural evaluations.

## Conclusion

This report highlights a case of Hashitoxicosis following RFA for PTMC in a healthy 48-year-old woman, emphasizing the potential for autoimmune hyperthyroidism as a post-RFA complication. These findings underscore the value of preprocedural thyroid antibody testing in identifying patients at risk and enhancing our understanding of autoimmune responses, particularly in the context of RFA for thyroid nodules.

## Ethics approval and consent to participate

Written informed consent form was given to patient.

## Consent for publication

Not applicable.

## Availability of data and materials

Availability of data and materials supporting our findings will be shared upon request.

## Author contributions

All authors contributed to data analysis, drafting or revising the article, have agreed on the journal to which the article will be submitted, gave final approval of the version to be published, and agree to be accountable for all aspects of the work.

## Patient consent

This statement confirms that the patient(s) fully understand the purpose of the publication and have voluntarily agreed to share their information. This consent must be documented to respect the patient's rights and maintain ethical standards in medical publishing.
